# Inferences Drawn from a Risk Assessment Compared Directly with a Randomized Trial of a Home Drinking Water Intervention

**DOI:** 10.1289/ehp.8682

**Published:** 2006-04-04

**Authors:** Joseph N.S. Eisenberg, Alan Hubbard, Timothy J. Wade, Matthew D. Sylvester, Mark W. LeChevallier, Deborah A. Levy, John M. Colford

**Affiliations:** 1 Department of Epidemiology, School of Public Health, University of Michigan, Ann Arbor, Michigan, USA; 2 Center for Occupational and Environmental Health and Division of Epidemiology and Environmental Health Sciences, School of Public Health, University of California–Berkeley, Berkeley, California, USA; 3 National Health and Environmental Effects Research Laboratory, Office of Research and Development, U.S. Environmental Protection Agency, Chapel Hill, North Carolina, USA; 4 American Water, Voorhees, New Jersey, USA; 5 Division of Healthcare Quality Promotion, National Center of Infectious Diseases, Centers for Disease Control and Prevention, Atlanta, Georgia, USA

**Keywords:** drinking water, gastrointestinal, intervention trial, microbial risk assessment, waterborne pathogens

## Abstract

Risk assessments and intervention trials have been used by the U.S. Environmental Protection Agency to estimate drinking water health risks. Seldom are both methods used concurrently. Between 2001 and 2003, illness data from a trial were collected simultaneously with exposure data, providing a unique opportunity to compare direct risk estimates of waterborne disease from the intervention trial with indirect estimates from a risk assessment. Comparing the group with water treatment (active) with that without water treatment (sham), the estimated annual attributable disease rate (cases per 10,000 persons per year) from the trial provided no evidence of a significantly elevated drinking water risk [attributable risk = −365 cases/year, sham minus active; 95% confidence interval (CI), −2,555 to 1,825]. The predicted mean rate of disease per 10,000 persons per person-year from the risk assessment was 13.9 (2.5, 97.5 percentiles: 1.6, 37.7) assuming 4 log removal due to viral disinfection and 5.5 (2.5, 97.5 percentiles: 1.4, 19.2) assuming 6 log removal. Risk assessments are important under conditions of low risk when estimates are difficult to attain from trials. In particular, this assessment pointed toward the importance of attaining site-specific treatment data and the clear need for a better understanding of viral removal by disinfection. Trials provide direct risk estimates, and the upper confidence limit estimates, even if not statistically significant, are informative about possible upper estimates of likely risk. These differences suggest that conclusions about waterborne disease risk may be strengthened by the joint use of these two approaches.

Continued reporting of outbreaks of disease from consumption of drinking water (Barwick et al. 2000; Lee et al. 2002; Levy et al. 1998; Yoder et al. 2004) in the United States has fueled the need for regulatory action through risk assessments as mandated by the Safe Drinking Water Act (SDWA 1996). Risk assessments historically have been used to evaluate the health risks of properly treated drinking water because of the general belief that drinking water risks were too low to be detected through epidemiology studies. Recent drinking water intervention trials, however, have begun to question the assumption that there is little or no risk of infectious gastrointestinal (GI) illness attributable to the consumption of drinking water when water treatment systems are functioning properly (Payment et al. 1991, 1997). In contrast, other trials have suggested that there is little or no risk (Colford et al. 2005; Hellard et al. 2001). Based on these findings and in response to the 1996 Congressional amendment to the SDWA that emphasizes the need for sound science and risk-based standard settings [U.S. Environmental Protection Agency (EPA) 1989], there has been increased interest in evaluating methodologies to help estimate the risk of GI illness attributable to drinking water in communities. In the present study we compare and contrast two approaches for the assessment of risk of diarrhea caused by drinking water—a microbial risk assessment and a randomized intervention trial design.

Using data collected in Davenport, Iowa (Colford et al. 2005), we compared the two techniques to estimate the risk from waterborne pathogens due to exposure to drinking water. For this study, risk assessment is based on the integration of several independent sources of exposure information to estimate dose (i.e., water quality, drinking water treatment plant efficiency, and tap water consumption patterns). We then used the dose information in a health effects model to predict the risk of illness due to drinking tap water. The randomized intervention trial directly measures the impact of drinking water on diarrhea and compares the incidence of GI illness between intervention and control subjects.

Both approaches have wide appeal. The randomized trial is considered the “gold standard” for providing unconfounded causal risk estimates associated with a particular exposure. When lacking these direct estimates of risk, quantitative risk assessment is the preferred method for attaining risk estimates and is used by the U.S. EPA, U.S. Food and Drug Administration, World Health Organization, and other stakeholders for regulatory and operational purposes. Although these approaches are widely accepted, they also have many limitations. Low sensitivity because of sample size constraints, and biases due to both exposure and outcome misclassification must be acknowledged when interpreting randomized trial results. Similarly, risk assessments are model-based estimates and rely on water quality data as input, and so must be interpreted in this context. Both approaches have their strengths and weaknesses. In the present study our goal was to compare and contrast the two approaches for obtaining estimates of drinking water risk when coincident data are available.

Several authors have proposed methods for estimating the risk of drinking water (Haas et al. 1993; Messner et al. 2001; Regli et al. 1999). Our study differs from these previous studies in that we incorporated additional detailed local information relevant to risk assessment, including measurements of pathogen levels in the source water over a 1-year period, pathogen removal efficiency of the Davenport drinking water treatment plant (which uses sedimentation, filtration, and chlorine disinfection), and data on local tap water consumption.

## Materials and Methods

### Attributable risk from intervention trial (Davenport, Iowa)

The study design of the intervention trial in Davenport is similar to those of previously published drinking water intervention trials (Colford et al. 2002; Hellard et al. 2001; Payment et al. 1991, 1997). Unlike prior randomized trials, however, a crossover design was used where, for each intervention period (~ 6 months), half the enrolled cohort had a water treatment device installed at their kitchen faucet and half had a sham device installed that resembled the real device but provided no water treatment. At the end of the first treatment period, the device in each subject’s household was switched to the opposite type, and illness was monitored for another 6 months. Participants were blinded throughout the study to their specific device type, and they recorded their daily occurrence of GI symptoms (e.g., diarrhea, nausea, vomiting, cramps) in a personal health diary. The study resulted in treatment assignment and illness data for 1,296 subjects in 456 households. For further details of the Davenport intervention trial, see Colford et al. (2005). As part of the Davenport intervention study, a separate random digit dial (RDD) telephone survey was conducted in the Davenport area. The goal of the survey was to obtain population-based estimates of the use of various home water treatments, water consumption, and the monthly occurrence of GI illnesses (Wade et al. 2004).

We define attributable risk (AR) for the trial subjects as the estimated risk difference in daily rates of highly credible GI illness (HCGI) (Colford et al. 2005) among the subjects with the treatment device versus those with the sham device. HCGI is defined as the presence of any one of the following syndromic manifestations of GI illness: vomiting, watery diarrhea, soft diarrhea with abdominal cramps, and nausea with abdominal cramps. The AR was estimated using a linear model with binomial errors and accounting for correlation using a generalized estimating equation (Zeger et al. 1988).

### Risk assessment model

**Figure 1:**
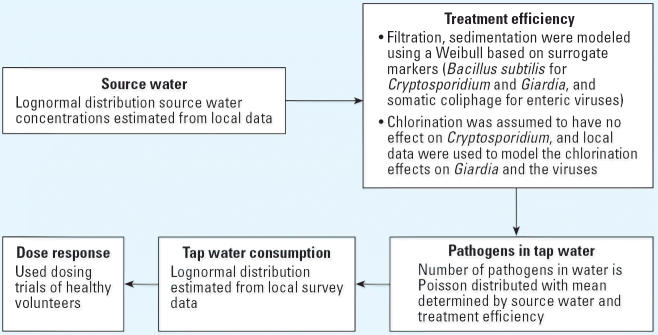
Schematic of risk model.

The risk assessment was conducted without knowledge of the results of the Davenport trial. Figure 1 is a schematic of the general model for generating GI illness cases due to drinking water. Methods used to derive the model parameters are discussed later in this article. The model uses a population of 10,000 and a risk period of 1 year (365 days). The model is a simple linear process and works as described below.

A concentration of the specific source water distribution of pathogens (e.g., *Giardia*, *Cryptosporidium*, and culturable viruses) is randomly sampled for the day. On the basis of previous studies and goodness-of-fit tests of the source water data collected in Davenport, we assumed that the average concentrations of source water for a day followed a lognormal distribution (LeChevallier et al. 2003b). This distribution was estimated using the constant recovery rates shown in Table 1.

We assumed that treatment efficiency due to sedimentation and filtration remained constant during the day but itself was a random draw from a Weibull distribution (Ross 1985). Similarly, the disinfection due to chlorine for *Giardia* and viruses was a random draw from a Weibull distribution. The Weibull distribution provided the needed flexibility to fit the various treatment data.

The concentration of pathogens in the resulting drinking water, *D_i_*, for day *i*, was


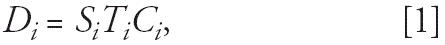


where *S_i_*, *T_i_*, and *C_i_* are the (daily) randomly drawn source water concentration, treatment efficiency, and disinfection, respectively.

For each day *i*, for each of 10,000 individuals *j*, we randomly drew a volume of water consumption, *V_ij_*, from a lognormal distribution (Rosebury and Burmaster 1992) based on data from the RDD telephone survey in Davenport (Wade et al. 2004).

A random number of pathogens, *P_ij_*, ingested for each subject *i*, on each day *j*, was generated from a Poisson distribution with mean, *V_ij_* × *D_i_*. We generated a random (yes/no) indicator of illness, *I_ij_*, based on the number of pathogens and the probability of illness given *P_ij_*. This probability was derived from separate dose–response curves (probability of infection for a given ingested pathogen dose) and morbidity ratios (the ratio of those who become ill to those who are infected) for each pathogen, which were based on published dose–response data (DuPont et al. 1995; Rendtroff 1954; Teunis et al. 1986; Ward et al. 1986).

The final step, after generating data for 10,000 subjects and 365 days, is to count the number of events and divide by the time at risk to derive an estimate of disease incidence due to exposure to the specific pathogen in drinking water for the year:


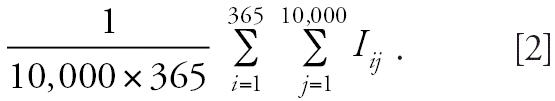


### Parameter estimates in risk assessment model

Each step of the above model relies on parameter estimates. We derived almost all of these estimates from site-specific (Davenport) data. When site-specific data were not available, we used data from the literature.

#### Source water concentration

Water quality data from the source water serving the study area came from the Davenport intervention study. These included approximately weekly measurements of *Cryptosporidium* and *Giardia* concentrations, as well as monthly measurements of culturable viruses (LeChevallier et al. 2003b).

**Figure 2:**
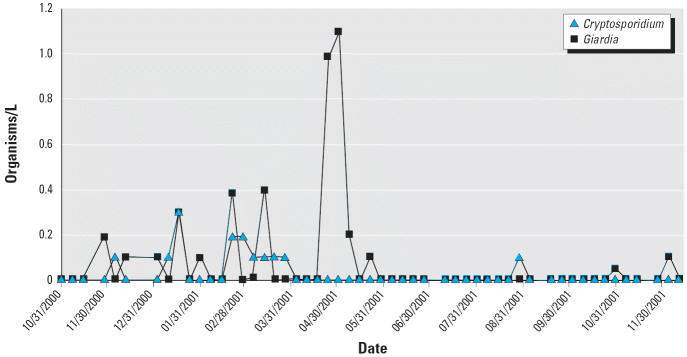
Raw water measurements of *Cryptosporidium* and *Giardia*.

**Figure 3:**
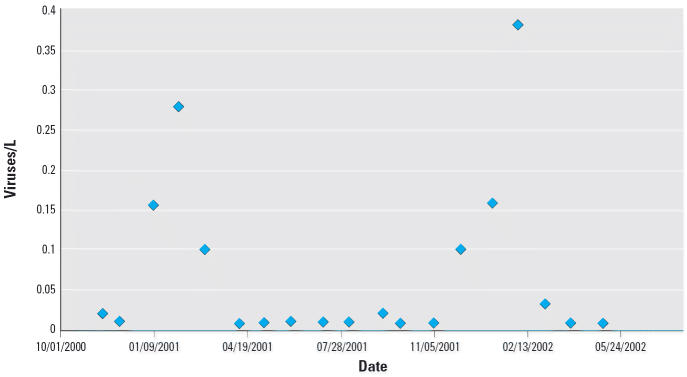
Raw water concentrations of total culturable enteric viruses (most probable concentration of viruses per liter).

Figure 2 shows the raw data for both *Giardia* and *Cryptosporidium*, *X_k_* , collected at different days, *k*, and Figure 3 shows similar data for the culturable viruses. These represent counts of pathogens in a fixed volume, *Q*, of sampled source water with assumed recovery rate, *R*. We assume that the counts of pathogens, *X_k_* , are derived from an underlying Poisson distribution with mean *S_k_* × *Q* × *R*, where *S_k_* is the average source water concentration for day *k*. We assumed that *S_k_* follows a lognormal distribution, suggesting that a marginal likelihood of *X* is


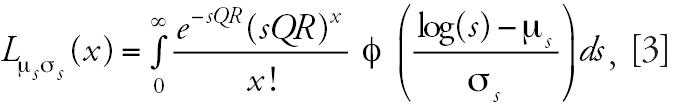


where φ represents the standard normal density, and μ*_s_* and σ*_s_* are the underlying mean and standard deviation, respectively, of the log(*S*) distribution. Equation 3 represents the likelihood contribution of one observation of the raw counts, *X_k_* ; the parameters of interest, μ*_s_* and σ*_s_*, are estimated by maximizing the likelihood. The estimates of all parameters in the model are presented in Table 1.

#### Treatment efficiency

Direct estimates of treatment efficiency with respect to *Cryptosporidium* species, *Giardia* species, and culturable viruses were not possible from the Davenport treatment facility because levels in effluent water samples were uniformly below detection across the study period. Estimates of the efficiency of *Bacillus subtilis* treatment, obtained from weekly measurements of source water and plant effluent data (LeChevallier et al. 2003b), were used as a surrogate for *Cryptosporidium* and *Giardia* treatment efficiency. Similarly, removal of somatic coliphage from waters passing through the Davenport facility was used to approximate a distribution of treatment efficiency with respect to culturable viruses.

The log removal for *Cryptosporidium* from chlorine disinfection was assumed to be zero, whereas the log removal for *Giardia* and enteric viruses was estimated from chlorine concentration time (CT) values collected in Davenport. The CT values were estimated for *Giardia* and were therefore directly applied to estimate disinfection efficacy for *Giardia*. Because there were no equivalent data for viruses, virus disinfection was assumed to have the same distribution as *Giardia* but a modulated mean value. One way to establish specific viral log removal values for the model is to rely on data presented in a U.S. EPA guidance document (U.S. EPA 1991). Table E-7 in this guidance document suggested that 4 log removal of viruses would be achieved at 20°C, a pH of 6–9, and a CT value of 3. This table was based on hepatitis A data (Sobsey 1988) assuming both a 3-fold safety factor and a 2-fold decrease in CT for every 10°C increase in temperature. Using Table E-4 from this same guidance document, we can estimate that the CT value in Davenport was approximately 13. Assuming a linear relationship between viral log removal and CT would suggest that the log removal of viruses by disinfection was > 12. This result assumes that viruses are dispersed in chlorine-demand–free water and is not valid for viruses that occur in nature aggregated and associated with organic particles. Given the uncertainties associated with all of these assumptions, we chose to examine a variety of viral log removal values ranging from 4, the minimum required by the U.S. EPA, to the 13 log removal treatment level estimated above.

#### Water consumption

During the period of the intervention trial, home tap water consumption data were collected from the RDD telephone survey. The estimate of the distribution of regular tap water consumption was obtained from 4,756 interviews. The water consumption distribution was assumed to be lognormal (Rosebury and Burmaster 1992), and we estimated the mean and standard deviation of this distribution. The RDD survey respondents were asked how much water was consumed in discrete glasses: < 1, 1–2, 3–5, and > 5. We took the number of respondents in each of these categories, made the categories contiguous (i.e., < 1, 1–2.5, 2.5–5, > 5), and estimated the mean and standard deviation of log consumption using maximum likelihood.

#### Probability of disease

The functions used to generate a probability of disease given a quantity of pathogen ingested (dose response) were derived from dosing trials where healthy volunteers were given known quantities of pathogens. Specifically, DuPont et al. (1995) published data for a sample of healthy volunteers infected by known numbers of *Cryptosporidium* oocysts, Rendtroff (1954) reported similar data for *Giardia*, and Ward et al. (1986) for rotavirus, which we used as a surrogate for culturable viruses (Regli et al. 1991). Exponential functions were used for *Giardia* and *Cryptosporidium*, and a beta-Poisson function for culturable enteric viruses (Teunis et al. 1996).

## Results

The estimated attributable (annual) rate of disease per 10,000 people from the Davenport trial (expressed as the rate in the sham group minus the rate in the active group) was −365 [95% confidence interval (CI), −2,555 to 1,825], which provided no evidence of a significant association of the use of drinking water with disease. The result was negative because there were more cases reported from the active than from the sham group. Based on the upper value of the 95% CI, the trial was statistically consistent with as many as 1,825 cases per 10,000 people per year attributable to drinking water. These estimates were calculated from a cohort of 1,296 persons that reported 394 episodes of HCGI while in the active group and 350 while in the sham group (Colford et al. 2005).

Table 2 is a summary of the estimated cases of illness from our risk assessment models based on pathogens. Assuming a 4 log removal of viruses from disinfection (U.S. EPA regulatory limit), the predicted risk was 13.9 (2.5, 97.5 percentiles: 1.6, 37.7) cases per 10,000 persons per year (due to *Cryptosporidium*, *Giardia*, and culturable enteric viruses), whereas assuming an 6 log removal, the predicted risk dropped to 5.5 (2.5, 97.5 percentiles: 1.4, 19.2) cases. At 6 log removal there was less than 1 case associated with viral exposure. Results from higher viral log removal did not vary from the results using 6 log removal. The width of the CI values from the Davenport trial and risk assessment should not be compared, as the former incorporates sources of variation and uncertainty that are not relevant in the latter.

We also examined the sensitivity of our risk assessment results to alternative parameterizations of the model by conducting the following sensitivity analyses: *a*) instead of assuming a Poisson distribution, we modeled pathogen density using a negative binomial distribution with different levels of aggregation; *b*) rather than using Davenport-specific treatment efficiency values, we used published values (Rachwal et al. 1996); *c*) rather than using site-specific data from the RDD telephone survey, we based the mean and standard deviation of the estimated average daily water consumption on reported U.S. EPA values (U.S. EPA 2000); and *d*) we varied the two dose–response parameters by a factor of 10. Results based on the above variations increased predicted *Cryptosporidium* cases to be as high as 25 cases per 10,000 persons per year, *Giardia* cases to as high as 100, and culturable enteric viruses to as high as 15. This brings the predicted risk to as high as 140 cases per 10,000 persons per year. There was little effect from adding overdispersion to the pathogen distribution using the negative binomial model. The higher estimates were primarily because of the use of non-Davenport-specific treatment values.

## Discussion

In this study, both risk assessments and intervention trials are used to obtain health risk estimates. The interpretation of the results obtained from these two approaches, however, can often differ. The data collected in Davenport provided a unique opportunity to compare and contrast these two approaches. Even though there was no evidence of a significant association in the Davenport analysis, the upper bound risk estimate from the intervention trial (based on the 95% CI) was higher than the drinking water standards provided by the U.S. EPA. Under these rigorous standards, the Davenport analysis provides a useful upper bound on the risk; however, a risk assessment is needed to estimate the risk within the limits set by regulatory agencies. Specifically for Davenport, the trial estimated an upper-end risk of 1,825 cases per 10,000 persons per year, whereas the risk assessment predicted 5–14 cases per 10,000 persons per year attributable to drinking water from *Giardia*, *Cryptosporidium*, and culturable viruses. An additional finding in our work was a difference in the estimation of illnesses provided by risk assessment when using site-specific water quality data rather than generally available estimates of treatment efficacies.

Because of the different approaches used by risk assessments and intervention trials, the analytic results from each approach often have different interpretations. These differences in the two approaches are summarized in Table 3 and discussed below.

### Sensitivity

Historically, drinking water regulations have been based on a tolerable annual risk of 1 case per 10,000 persons, that is, a goal of fewer than one case of infection with a particular pathogen per 10,000 persons attributable to drinking water (Regli et al. 1999). Although this value is not explicitly used by the U.S. EPA, it is consistent with their regulatory guidelines (Regli et al. 1999). Epidemiologic studies, such as the intervention trial conducted in Davenport, generally cannot measure such low-magnitude risks. The Davenport trial was powered to detect approximately 1,100 illnesses per 10,000 persons—a smaller risk difference than that observed in previous studies (Payment et al. 1991). To illustrate this lack of sensitivity, we estimated that to power the Davenport trial to detect an AR of 20 cases per 10,000 persons per year, a risk similar to that estimated by the risk assessment, would require a sample size of 8 million individuals; to detect an AR of 100 cases per 10,000 persons would require 416,000 individuals. The intervention trial, using traditional levels of statistical significance, lacks the sensitivity to detect the low number of cases predicted from the risk assessment.

In addition to a limited sample size, the sensitivity of a trial may be decreased because of biases caused by, for example, misclassification of disease outcomes and exposure (i.e., people with disease are more or less likely to change their drinking water patterns). Because of randomization, most misclassification in the trial was likely to be nondifferential; that is, if subjects are underreporting disease, they are likely to be doing so equally while in the both the active treatment and the sham group. Because nondifferential misclassification biases the estimate of AR toward the null (Rothman and Greenland 1998), estimates that do not account for this misclassification (e.g., the estimate in this study) would likely underestimate the magnitude of these estimates. Adjusted risk estimates would increase both point estimates and the upper end of the 95% CI.

Even if the sensitivity of the intervention trial precludes us from making any inference within the narrow range of regulatory limits, the trial data do provide a rigorous direct estimate of the upper bound to the risk from drinking water. For example, previous drinking water intervention trials in Canada showed that up to 35% of GI illnesses were transmitted through a public drinking water system (Payment et al. 1991). The Davenport intervention study was adequately powered to detect differences considerably smaller than this level and clearly demonstrates that the drinking water risks estimated in Davenport were below those observed by Payment (1991); that is, the upper end of the 95% CI for the percent AR reported by Colford et al. (2005) was 10%, whereas the point estimate reported by Payment et al. (1991) was 35%. For regulatory purposes the upper bound estimate from Davenport can be interpreted as the largest risk estimate that is still consistent with the intervention trial results. As mentioned above, this upper bound estimate is not only based on random error, due to sample size, but also on systematic error in a trial, such as the biases due to nondifferential misclassification.

### Causal evidence and pathogen-specific versus symptomatic outcomes

Risk assessment methods also have limitations. For example, risk estimates are model based and provide indirect evidence of risk based on water quality data, whereas the trials focus on direct estimates of illness. Additionally, the risk estimates include only a subset of the potential pathogens, compared with the intervention trial, which provides a risk estimate for diarrheal disease integrated over all pathogens as well as nonpathogenic causes of diarrhea. In this risk assessment we were able to provide estimates for two protozoan pathogens, *Cryptosporidium* and *Giardia*, as well as for culturable viruses. The microbiologic methods, however, identify all species of *Cryptosporidium* and *Giardia*, not all of which cause illness. Likewise, viruses that are culturable are only a subset of known viral pathogens; for example, noroviruses are a major cause of waterborne viral infections but are not culturable. These limitations can therefore lead to both under-and overestimates of risk.

### Model specification and the inclusion of other sources of risk

The specific model structure used in any risk assessment carries with it many assumptions. For example, the model in this study was based on risks associated with source water contamination and did not consider the potential contamination within the distribution system (LeChevallier et al. 2003a). Another assumption implicit in the model structure is that secondary transmission is negligible (Eisenberg et al. 2002, 2003). Both of these assumptions can lead to biased results. By incorporating such processes, risk assessment models can be made more complicated and perhaps more accurate. Some environmental processes, such as risk incurred by the distribution system, can be also captured by the intervention trial and in fact was accounted for in the Davenport trial. Other processes, such as transmission processes, can be addressed only in a limited way by observing within-household transmission. Specifically, the standard intervention trial design is focused on individual-level risk, assuming that disease outcomes of different individuals are independent and therefore cannot capture population-level processes such as secondary transmission. Risk assessment models that incorporate disease transmission processes are the only models that account for these population-level conditions. Increasing the complexity of the model, however, leads to additional uncertainty with respect to model specification and potentially parameter specification.

In general, it is impossible to account for all sources of variation. Models often must rely on estimates from small studies (e.g., dosing trials conducted on healthy individuals) and on very strong modeling assumptions. Sparse site-specific data, such as with the source water measurements of enteric viruses, increase the uncertainty of model-based estimates.

Most of these limitations underestimate risk. There are additional uncertainties that result in overestimates of risk. For example, uncertainties in treatment—*Bacillus* spores are considered conservative indicators of *Cryptosporidium* removal, and disinfection CT values are based on half-lives, not the full integration of disinfection contact times.

### Site-specific versus general estimates in risk assessment

Our finding of a difference in estimates of illness from using site-specific rather than general U.S. EPA estimates for treatment efficiencies highlights the importance of a clear definition for a risk assessment. If the goal of a risk assessment is focused only on risk within a specified community (or similar communities), then site-specific data may be most appropriate and worth the additional effort to obtain. If, however, the goal is to generalize about risk across multiple communities or large areas, then the general parameter estimates provided by the U.S. EPA are likely to be more appropriate.

### Examining alternative control strategies

One advantage of a model-based risk assessment is that alternative control strategies can be examined. For example, the pathogen-specific risk estimates from the risk assessment provided additional information for focused waterborne disease control strategies. Given that the viral log removal by disinfection is on the order of 6 or more, the predicted risk above 1 in 10,000 persons comes from exposure to protozoan species. Thus, if the risk levels presented in Table 2 are of concern, control efforts should be focused on treatment technologies that address protozoa rather than virus removal. If, on the other hand, viral log removal by disinfection is ≤ 4, control efforts should be focused on treatment technologies that address viral removal. Given the limited data to inform this assumption, resources could be focused on collecting more viral disinfection data.

### Cost and time

An additional limitation of intervention trials is that they are costly and time-consuming to conduct. In contrast, risk assessments are relatively inexpensive and quick to conduct.

## Conclusions

Risk assessment and intervention trials provide complementary approaches to the estimation of a community’s burden of disease attributable to drinking water. Risk assessments can provide estimates of low-risk situations; require data that are neither difficult nor expensive to collect; permit the evaluation of scenarios outside the conditions under which the data were collected and are therefore an attractive method for characterizing both existing and potential risk from contamination of drinking water; and can capture population-level processes such as secondary transmission. Intervention trials provide direct measures of AR within communities and provide risk estimates based on all causes of illness attributable to the drinking water. Even when point estimates of risk are not significant, these direct measures of risk can provide valuable upper bound estimates.

Given their expense, intervention trials must be judiciously applied. Risk assessments can be used to specify the conditions in which future trials are justified; that is, they can be used to identify high-risk conditions based on demographics, magnitude and sources of environmental contamination, and types of treatment processes. Risk assessment can also provide information on where are the important data gaps. In particular, this assessment pointed toward the importance of attaining site-specific treatment data and the clear need for a better understanding of viral removal by disinfection. Ultimately, the choice of risk assessment, intervention trials, or both methods used jointly to evaluate waterborne disease risks depends upon specific research needs and available funding.

## Figures and Tables

**Table 1:** Values used for risk assessment models for the different pathogens.

	Model		
Model component	*Cryptosporidium*	*Giardia*	Viruses
Source water			
Concentration (organisms/L, mean ± SD)^a^	1.06 ± 2.24	2.68 ± 24.20	0.93 ± 3.00
Recovery rate^b^	0.40	0.40	0.48
Treatment efficiency (log removal)			
Sedimentation and filtration (mean ± SD)^a^	3.84 ± 0.59	3.84 ± 0.59	1.99 ± 0.52
Chlorination (mean ± SD)^a^	0	3.5 ± 2.93	4 ± 2.93
Water consumption (L/day, mean ± SD)^c^	1.2 ± 1.2	1.2 ± 1.2	1.2 ± 1.2
Dose response^d^	λ = 0.004078	λ = 0.01982	α, β‚ = 0.26, 0.42
Morbidity ratio^e^	0.39	0.40	0.57

Sample mean ± SD values are reported.

a Where λ was estimated using data from DuPont et al. (1995) and Rendtroff (1954), respectively. Estimates using data collected in Davenport (LeChevallier et al. 2003b). All source water data were modeled using a lognormal distribution. A Weibull distribution was used for all treatment data. Disinfection for *Cryptosporidium* was assumed to be zero.

b Where α and β were estimated using data from Ward et al. (1986). Fraction of pathogens recovered. Data were from the Information Collection Rule Supplemental Survey (U.S. EPA 2001) after eliminating extreme observations (i.e., some samples reported a recovery rate > 100% or < 0%).

c Consumption of untreated water based on data from an RDD survey conducted in parallel with the trial. All pathogen models used the same lognormal distribution.

d The *Cryptosporidium* and *Giardia* dose–response models used an exponential function [Pr(*D*|*X*) = 1 − exp(−λ*X*)] where λ was identified using data from DuPont et al. (1995) and Rendtroff et al. (1954), respectively. The rotavirus dose–response model used a beta-Poisson function [Pr(*D*|*X*) = 1 − [1 + (*X*/β)]^−α^] where α and β were identified using data from Ward et al. (1986). *D* is disease, and *X* is dose.

e The ratio of those who become ill to those who are infected: *Cryptosporidium* (DuPont et al. 1995), *Giardia* (Nash et al. 1987), and viruses (Ward et al. 1986).

**Table 2:** Illness risk estimates associated with drinking water (cases per 10,000 persons per year) predicted by the risk assessment model.

	Cases of illness	
Pathogen	Mean	2.5–97.5 Percentile range
*Cryptosporidium*	2.1	0.8–3.5
*Giardia*	3.4	0.6–15.5
Enteric viruses^a^	8.4	0.2–18.7
Enteric viruses^b^	0	0–0.2

The percentile reflects the variability of the predicted mean estimate.

a Assumes that disinfection results in a 4 log removal.

b Assumes that disinfection results in a 6 log removal.

**Table 3:** Comparison of methodologic considerations between drinking water risk assessment models and intervention trials.

Methodologic considerations	Risk assessment	Intervention trials
Sensitivity	Not relevant	Low
Causal evidence	Indirect	Direct
Pathogen inclusion	Few	Many
Model specification	Adds uncertainty	Not relevant
Transmission processes	Can be included^a^	Only in a limited way
Distribution system effects	Can be included^a^	Was included
Examining alternative control strategies	Yes	No
Expense	Low	High
Time	Fast	Slow

a Not included in this study.
